# Ten years of leadless pacing

**DOI:** 10.1093/eurheartjsupp/suae102

**Published:** 2025-03-24

**Authors:** Laurence M Epstein, Mikhael F El-Chami, Clemens Steinwender, Kyoko Soejima

**Affiliations:** Northwell Health, New Hyde Park, NY, USA; Emory University, Atlanta, GA, USA; Department of Cardiology, Kepler University Hospital, Medical Faculty, Johannes Kepler University, Linz, Austria; Kyorin University Hospital, Tokyo, Japan

**Keywords:** Leadless pacemaker, Pacemaker, Heart block

## Abstract

Pacemakers are a lifesaving and mature technology. The past decade has seen a revolution in pacemaker technology with the development of leadless pacemakers. There is growing evidence for the value of leadless pacemakers in the management of a variety of patients.

## Introduction

The development of implantable pacemakers has been a truly life-changing technology for millions of patients since the first human implant in 1958.^1^ Advances over the subsequent decades have resulted in programmable, dual chamber, and biventricular devices, which are significantly smaller and longer lasting. Despite these advances, transvenous pacemakers are far from perfect. Lead- and pocket-related issues remain the weak links of transvenous pacing.^2^ In addition to implant-related complications (pneumothorax and perforation), transvenous leads are associated with lead failure, venous occlusion, tricuspid regurgitation, and potential infectious seeding.^3,4^ Pacemaker pockets are subject to both acute and more often delayed infection, especially following generator changes.^5^ Lastly, the presence of the pocket can lead to patient discomfort, both physical and psychologic. These issues led to the desire to develop a leadless pacing system.

## Leadless pacing

**Figure 1:**
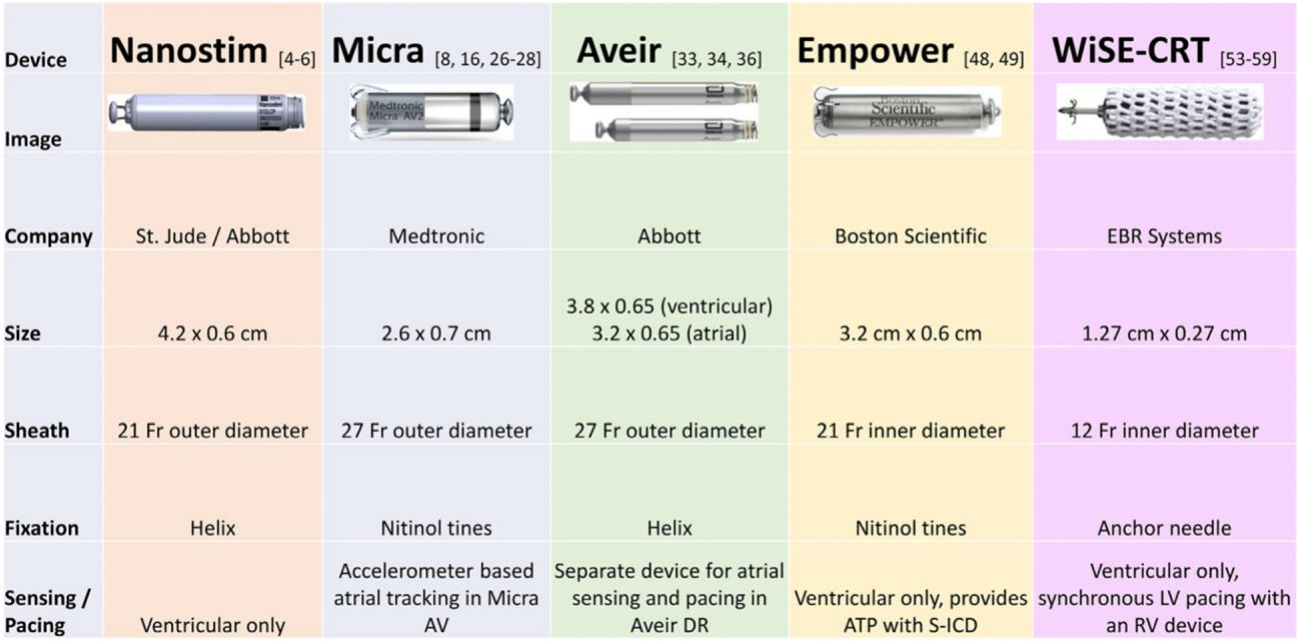
Leadless pacemakers and characteristics (Reprinted from *Trends Cardiovasc Med.*, Volume 34; Beccarino N, Saleh M, Epstein LM; Leadless pacemakers: Where are we?; pages 531-538; Copyright 2024, with permission from Elsevier.).

The concept of a completely intracardiac pacemaker was first introduced in 1970.^6^ Leadless pacemakers (LP), however, did not mature into a pacing therapy until December 2012 with the implantation of the Nanostim (St. Jude Medical Inc., Saint Paul, MN, USA; now Abbott Medical Inc., Abbott Park, IL, USA) and in 2013 with the implantation of the Micra (Transcatheter Pacing System) TPS (Medtronic, Minneapolis, MN, USA) as part of investigational device exemption (IDE) studies. The Nanostim received CE Mark in October of 2013. However, due to battery-related issues and reports of docking button detachment, the device was removed from the market in October 2016.^7^ The next generation of the Nanostim, the Aveir LP (Abbott, Abbott Park, IL, USA) received Food and Drug Administration (FDA) approval in 2022. Micra VR received CE Mark in 2015 and FDA approval in 2016. In addition to the approved leadless devices, others are in investigation (*Figure 1*). This review will be limited to the currently approved LPs. The trials reviewing the important clinical trials of the commercially available leadless pacemakers can be found in *Table 1*.

**Table 1:** Clinical trials of commercially available leadless pacemakers

Study	Authors	Device	Sample	Result	Complications
A Leadless Intracardiac Transcatheter Pacing System	Reynolds, et al.^8^	Micra VR	725	99.2% rate of successful implantation. 98.3% rate of acceptable capture threshold at 6 months.	96% freedom from major complications at 6 months. Fewer complications than previously described transvenous controls.
Micra Coverage with Evidence Development study	El-Chami, et al.^16^	Micra VR	6,219	Fewer re-interventions in Micra patients relative to a cohort of traditional pacemakers. Hazard ratio (HR) 0.62, 95% confidence interval (CI) 0.45–0.85, P=0.003.	Fewer chronic micra complications relative to a cohort of traditional pacemakers (adjusted HR 0.69, 95% CI 0.60–0.81, P<0.0001).
Micra Post-Approval Registry	El-Chami, et al.^15^	Micra VR	1,809	99.1% rate of successful implantation. 2% rate of CRT upgrades through 5 years.	4.5% major complication rate at 5 years
MARVEL	Chinitz, et al.^26^	Micra AV	64	After download of AV synchrony algorithm into previously placed Micra devices, average AV synchronous pacing was 87.0% (95% confidence interval 81.8%–90.9%).	No complications reported.
MARVEL 2	Steinwender, et al.^27^	Micra AV	75	After download of AV synchrony algorithm into previously placed Micra devices, there was an increase in AV synchronous pacing from 26.8% during VVI pacing to 89.2% during VDD pacing.	No episodes of pauses or pacemaker induced tachycardias noted.
AccelAV	Chinitz, et al.^28^	Micra AV	152	Resting AV synchrony was 85.4% at 1 month. Among those with programming optimization, AV synchrony improved 10.5% (P<0.001). Significant improvement in quality of life was noted (P−0.031).	There were no upgrades required to dual chamber or biventricular devices.
Micra AV Post-Approval Registry	Garweg, et al.^29^	Micra AV	801	Risk for major complications lower with Micra AV vs transvenous pacemakers (HR: 0.42; 95% CI: 0.28–0.61). 0.3% rate of system revisions due to pacemaker syndrome at 1 year.	3.7% rate of major complications at 1 year
LEADLESS II-Phase 2	Reddy, et al.^19^	Aveir VR	200	Freedom from complication as well as the effectiveness criteria of capture threshold and R wave amplitude were both met in 96% of patients.	Perforation and premature deployment both occurred in 1.5% of cases.
A dual-chamber leadless pacemaker	Knops, et al.^21^	Aveir DR	300	Successful dual-chamber implantation occurred in 98.3% of cases. Adequate capture and sensing thresholds were met in 90.2% of the patients and at least 70% atrioventricular synchrony was achieved in 97.3% of the patients.	The 90-day complication rate was 9.7%, including two pericardial effusions related to the atrial device and six dislodgements.

Adapted from *Trends Cardiovasc Med.*, Volume 34; Beccarino N, Saleh M, Epstein LM; Leadless pacemakers: Where are we?; pages 531–538; Copyright 2024, with permission from Elsevier.

## Micra TPS

The Micra TPS measures 2.6 × 0.7 cm (*Figure 1*) and is implanted in the right ventricle via a 27 Fr introducer sheath. Passive fixation nitinol tines engage the right ventricular trabeculations for fixation. The Micra TPS received FDA and CE Mark approval in 2016 and 2015, respectively, based on the data from the IDE study.^8^ The Micra post-approval registry (PAR) later confirmed the data seen in the IDE study and established LP as an alternative to the traditional transvenous pacemakers (TV-PPM).^9^ The Micra AV was FDA approved in 2020 as a single device that can track atrial mechanical contraction providing AV synchrony.^10,11^

Multiple studies have confirmed the safety and effectiveness of Micra TPS. The IDE study enrolled 726 patients.^8^ Implantation success rate was >99%, and the rate of major complications at 6 months was 4%. These major adverse events included a perforation rate of 1.6% and groin complications of 0.7%. No reported macro-dislodgment or infections were seen. These results were reassuring with some concerns about the rate of pericardial effusion. The Micra PAR is an FDA-mandated study that was designed to monitor the performance of this new device after its market release.^9^ The study enrolled 1817 patients with a reported implant success rate of 99.1% and total complication rate of 2.7% at 1 year. The rate of macro-dislodgment (0.06%), groin complications (0.6%), and infection (0%) was encouraging and replicated the IDE findings. In this study, a lower rate of pericardial effusion was seen (0.8% total and 0.4% meeting the definition of major complications). This was probably a reflection of more experience with this technology and targeting the interventricular septum for implantation rather than the apex. The Micra IDE and PAR included a historical cohort of patients implanted with TV-PPM. Both studies showed reduction in complication with a LP compared with TV-PPM (63% reduction in the PAR).

The Center of Medicare and Medicaid Services required enrolment of all Medicare recipients in a prospective study as a condition of coverage [the Coverage with Evidence Development (CED)]. In the Micra CED study, all Medicare patients receiving a Micra LP and single-chamber TV-PPM were enrolled, and the outcomes were analysed using claim data.^12^ The perioperative and long-term outcomes of >5700 Micra patients and 9662 TV-PPM were analysed. Perioperative complications were similar (7.7% vs. 7.4%, *P* = ns); LP was associated with a higher rate of pericardial effusion as compared with TV-PPM (0.8% vs. 0.4%, *P* < 0.05) but lower rate of device-related complications (1.4% vs. 2.5%, *P* < 0.05). The benefit of LP was apparent during intermediate-term follow-up. The 2- and 3-year follow-up of the Micra CED study showed a >30% reduction in total complications as well as a lower rate of re-intervention (≅40%).^13,14^ The 5-year follow-up of the Micra PAR was also recently published, showing a 53% reduction in total complications and a 53% reduction in the need for a system revision.^15^ These intermediate and long-term follow-ups from the CED and PAR studies highlight the benefits of LP over the long term and stress that the main benefit of this pacing technology is due to reduction in lead- and pocket-related complications.

## Micra AV

The Micra AV is considered an intermediate step between a single-chamber and dual-chamber LP. Using the 3 axis accelerometer signals that were originally designed to optimize rate response, engineers noted that these signals correspond to stages of the cardiac cycles. One of the signals recorded is the A4, which correlates with mechanical atrial contraction. Tracking A4 could allow for AV synchrony.^16^ The Micra AV has limitations when it comes to ensuring high degree of AV synchrony especially at faster heart rates and loses the ability to do so with heart rates above >110 b.p.m. This occurs due to the merging of the different cardiac signals. In addition at faster rates, the A4 signal might fall in the blanking period.^16^ However, this mode might be beneficial for the typical elderly patients with complete heart block where AV synchrony at fast heart rate is not essential. Other patients, such as young patients or patients where AV synchrony is important during exercise, may not be well served with this device. The MARVEL 2 study showed that the Micra AV algorithm results in a resting mean AV synchrony of 89%.^11^ The ACCEL AV study showed a sustained resting AV synchrony of 85% while ambulatory AV synchrony decreased to 75%.^10^ The group of patients that underwent AV optimization had a 10% increase in ambulatory AV synchrony. This highlights the need for routine optimization of AV synchrony in patients with Micra AV.

The Micra AV PAR results were recently published.^15^ This study enrolled 801 patients with an implant success rate of 99.4%, a perforation rate of 1.2%, and groin complications of 0.9%. No infections and dislodgment were noted. When compared with historical cohort of dual-chamber TV-PPM, there was 58% reduction in total complications and a lower rate of system revision (75% reduction as compared with TV-PPM).

## Micra VR2 and AV2

The next generation of the Mica, the Micra VR2 and AV2 is similar in dimensions and delivery. To address concerns over cardiac perforation, the delivery sheath was redesigned. The ‘cup’ tip was rounded and the surface area increased to reduce pressure against the cardiac wall.^17^ In addition, the battery was redesigned to improve longevity and the upper tracking rate limit for Micra AV2 was increased to 135 b.p.m.^18^

## Aveir LP

The ventricular Avier LP (Abbott, Abbott Park, IL, USA) is the revised version of its predecessor (Nanostim) and measures 3.8 × 0.65 cm. It employs an active fixation helix and is also delivered via a 27 Fr sheath. The ventricular Aveir LP received FDA approval in 2022 based on the LEADLESS II PHASE 2 study.^19^ The LEADLESS II Phase 2 study^19^ enrolled 200 patients. The implant success rate was 98%, and the total complication rate was 4% including pericardial effusion in 1.5% and device migration in 1%. A comparison of the efficacy and safety of the Aveir vs. the Micra found comparable efficacy and safety. There was a higher incidence of ventricular arrhythmia and longer procedural duration with the Aveir.^20^

Similar to the Micra, the Aveir Single-Chamber LP CED Post-Approval Study is currently ongoing to evaluate the long-term safety and efficacy of this device.

## Dual-chamber AVEIR LP

The dual-chamber Aveir LP (the atrial device measures 3.2 × 0.65 cm) received FDA approval in 2023 and CE Mark approval in 2024.^21^ The atrial and ventricular devices communicate using ‘implant-to-implant’ (i2i) communication. To preserve battery, a low-amplitude electrical signal conducts through the blood and myocardial tissue. The Aveir DR i2i study included 300 patients.^21^ The implant success rate was 98%. The rate of pericardial effusion was 0.7%, but the atrial dislodgment rate was concerning (1.7% intra-procedurally and 1.7% post-operatively). This might reflect early experience with this technology and will probably improve with experience. Endpoints of adequate capture and sensing thresholds were met in 90.2% of the patients, and at least 70% atrioventricular synchrony was achieved in 97.3% of the patients.

## Leadless pacing and infection

One of the weaknesses of transvenous pacemakers is infection, both pocket and systemic. Therefore, not surprisingly one of the goals for leadless pacing was a reduction in infection. In clinical practice, the incidence of infections related to ventricular LPs is limited to case reports. A review of El-Chami *et al*.^22^ highlights the reasons LPs may be infection resistant: While pacemaker implantation is a sterile procedure, studies have demonstrated that the ‘sterile’ gloves could be contaminated. There is no to minimal contact with the LPs during the implant procedure as opposed to the TV-PPM where contact with the leads and the generator is required. In addition, the absence of a subcutaneous pocket eliminates/reduces the risk of bacterial translocation through the skin. Also, the smaller device and lack of leads reduce the overall surface area that could be exposed to bacterial seeding. The Micra LPs are coated with parylene, a material that has antimicrobial properties. Lastly, ventricular LPs are in a high, turbulent flow area, reducing the chance of device seeding. Whether atrial leadless devices are as infection resistant, given the lower flow environment, is yet to be determined.

Given their infection resistance, LPs have been found to beneficial in pacemaker-dependent patients undergoing lead extraction for infection, obviating the need for temporary pacing.^23^

## Cost consideration

New technology is frequently more costly than the traditional technology that it is attempting to replace or complement. A prime example of this is LP. Whether the additional cost of these new devices will be offset by the intermediate and long-term risk reductions is not known. A study from Australia suggested that LPs are likely to be a cost-saving alternative to the traditional transvenous pacemakers.^24^

## The role of leadless pacing

At present, there are limited recommendations pertaining to LPs in societal guideline documents. The 2018 ACC/AHA/HRS document states ‘Identifying patient populations that will benefit the most from emerging pacing technologies (e.g. His bundle pacing, transcatheter leadless pacing systems) will require further investigation as these modalities are incorporated into clinical practice’, as most of the above data were not available at the time of writing.^25^ The 2021 ESC guidelines offer a 2A indication for patients with limited or no vascular access, or a high infectious risk, and a 2B indication as an alternative to standard single-chamber pacemakers.^26^ With the development of additional data and technologies to provide AV synchrony, a guidelines update is warranted.

As with most things in medicine, the choice of device type must be individualized. The advantages of LPs have been reviewed above. In patients with a reduced ejection fraction or the risk of developing pacing-induced myopathy, physiologic pacing has been demonstrated to be beneficial.^27^ The ‘Holy Grail’ and future of pacing is a leadless physiologic pacemaker, which is currently being explored.

## Data Availability

No new data were generated or analysed in support of this research.
